# Therapeutic potential of *Capparis spinosa* in experimental model of acute acetic acid-induced colitis: Anti-inflammatory and antioxidant effects

**DOI:** 10.1016/j.heliyon.2024.e32836

**Published:** 2024-06-13

**Authors:** Faezeh HosseiniRavesh, AmirAli Moodi Ghalibaf, Vahid Reza Askari, Farzad Fayedeh, Vafa Baradaran Rahimi, Leila Etemad, Zhila Taherzadeh

**Affiliations:** a Department of Pharmacodynamy and Toxicology, School of Pharmacy, Mashhad University of Medical Sciences, Mashhad, Iran; b Student Research Committee, Birjand University of Medical Sciences, Birjand, Iran; c Pharmacological Research Center of Medicinal Plants, Mashhad University of Medical Sciences, Mashhad, Iran; d Department of Cardiovascular Diseases, Faculty of Medicine, Mashhad University of Medical Sciences, Mashhad, Iran; e Medical Toxicology Research Center, Faculty of Medicine, Mashhad University of Medical Sciences, Mashhad, Iran; f Applied Biomedical Research Center, Mashhad University of Medical Sciences, Mashhad, Iran; g Targeted Drug Delivery Research Center, Mashhad University of Medical Sciences, Mashhad, Iran

**Keywords:** *Capparis spinosa*, Acute colitis, Anti-inflammation, Inflammatory cytokines, Tissue repair

## Abstract

**Introduction:**

This study examined the anti-inflammatory and antioxidant properties of *Capparis spinosa* L. (caper) in order to determine its medicinal potential in the treatment of acute colitis.

**Method:**

Sixty male rats were divided into six groups. After the experimental period, distal colonic extension was collected for determination of colonic damage, oxidative stress markers, along with antioxidant markers. The impact of altered levels of inflammatory cytokines in colon tissues on the underlying mechanisms examined.

**Results:**

The results showed that administering different doses of caper led to significant decreases in TNF-α and IL-6 levels when compared to the control colitis group (p < 0.001). Caper treatment effectively lowered elevated oxidative stress factors (MDA, NO, and MPO) compared to the control colitis group (p < 0.001). Caper treatment resulted in a significant increase in antioxidant factors (CAT, SOD, and GSH) compared with the control colitis group (p < 0.001).

Significant improvements in tissue repair were observed in caper-treated groups compared to positives and control colitis (p < 0.001).

**Conclusion:**

The study highlights caper may be useful in the treatment of acute colitis due to its ameliorative effects on inflammation, oxidative stress, and tissue repair.

## Abbreviation

3-(4,5-Dimethylthiazol-2-yl: MTT
5,5′-dithiobis-(2-nitrobenzoic acid): DTNB
Catalase: CAT
Cyclooxygenase: COX
Dimethyl sulfoxide: DMSO
Enzyme-linked immunosorbent assay: ELISA
Glutathione Peroxidase: GSH-Px
Total glutathione: GSH
Hydrochloric acid: HCl
Hydrogen Peroxide: H2O2
Inducible nitric oxide synthase: iNOS
Inflammatory bowel disease: IBD
inter quartile range: IQR
Interferon gamma: IFN-γ
Interleukin-6: IL-6
Interleukin-10: IL-10
Interleukin-13: IL-13
Interleukin-33: IL-33
Interleukin-37: IL-37
Janus kinase inhibitors: Jaki
Malondialdehyde: MDA
Myeloperoxidase: MPO
Nitric oxide: NO
Periodic Acid Schiff Staining: PAS
Reactive nitrogen species: NOS
Reactive oxygen species: ROS
Superoxide dismutase: SOD
Thiobarbituric acid: TBA
Trichloroacetic acid: TCA
Tumor necrosis factor receptor: TNFR
Tumor necrosis factor-α: TNF-α
Ulcerative colitis: UC

## 1 Introduction

Ulcerative colitis (UC) and Crohn's disease, often classified as inflammatory bowel disease (IBD), are a set of chronic gastrointestinal immune-mediated disorders with inflammation of rectal and colonic mucous [1]. UC is found to have the highest prevalence in North America and Europe, varying from 156 to 291 cases per 100,000 people-years. The prevalence is increasing owing to population aging in many industrialized countries across Africa, Asia, and South America [[2], [3], [4]]. Studies have shown higher rates of colorectal cancer among patients with chronic Crohn's disease and chronic ulcerative colitis because of chronic inflammation [5]. Alongside neoplasia, UC patients also suffer from a variety of complications, such as rectal abscesses, malnutrition, bone loss, thrombosis, and even some life-threatening issues, namely perforation and toxic megacolon [[6], [7], [8]].

Dysregulation of the inflammatory response has been found to be a key element in the pathogenesis of UC. NF-κB is commonly activated in patients with IBD and is known as the most prominent pathway for cell activation and secretion of several inflammatory and pro-inflammatory cytokines, such as IL-1, IL-6, and TNF-α [9]. Enhanced levels of TNF in IBD patients also further activate NF-κB through binding to tumor necrosis factor receptor 1 (TNFR1) and TNFR2 (Stillie & Stadnyk, 2009). Once activated it leads to transcriptional upregulation of several cytokines, chemokine, adhesion molecules, and enzymes, including IL-1, IL-6, TNF-α, COX-2, and iNOS, that are involved in the inflammatory cascade and recruitment of immune cells to the site of inflammation (Kim et al., 2007 [9]).

Pro-inflammatory cytokines such as IL-1, IL-6, IL-13, IL-33, and TNF-α have been shown to advance inflammation and anti-inflammatory cytokines including TGF-β, IL-10, and IL-37 have been shown to attenuate UC progression (Tatiya-aphiradee et al., 2019).

Oxidative stress is also another potential driving mechanism in the development and progression of UC through a build of ROS [10]. It has been shown that several cytokines, including TNF-α, IL-1, and interferon-which are highly increased in UC patients-, can cause the production of ROS. Lipid peroxidation is the process of free radicals reacting with lipids in the cell membrane, which results in the production of oxidized lipids (Cejas et al., 2004). It has been hypothesized that lipid peroxidation leads to crypt abscess propagation, and treatments targeting it, namely antioxidants are useful in controlling UC (Babbs, 1992; Bhaskar et al., 1995).

Corticosteroids, salicylates, anti-adhesion molecules, antibodies against TNF-α and integrin, and Janus kinase inhibitors (JAki) are some medicines that have been made to treat UC [[11], [12], [13], [14], [15]]. Even though the recommended medicines have been shown to help with treatment, they come with a lot of problems, including serious side effects, issues with the immune system, and issues that depend on how long you take them and how much you take [16]. Because of this, more research is needed to find medicines that are both effective and safe that can either treat or cure UC.

Complementary and alternative medicine can offer potential treatment choices along with standard care [17]. A growing body of research has shown success in the treatment of UC by many natural compounds including alkaloids [18], phenolic compounds [19], tannins [20], and flavonoids [21].

The mechanism by which these herbs work is to inhibit inflammatory pathways, such as NF-κB, COX, IFN-γ, and TNF-α [[22], [23], [24]]. These plants also attenuate oxidative stress. Reactive oxygen species (ROS) and nitrogen species (NOS), which are made when oxidative stress happens, have been linked to intestinal inflammation [23,25]. Because of this, herbal medicine seems to be a good way to treat UC, either instead of or in addition to other treatments.

Capparis spinosa L. is a plant that contains many natural and chemical compounds and has gained success in the treatment of many disorders (Annaz et al., 2022) [26,27]. Capparis spinosa L., widely known as caper, belongs to the Capparaceae family and is a perennial species mostly distributed in tropical regions [28]. According to existing research, multiple studies have shown the many advantages of capers, including its antioxidant [29,30], anti-tumor [30,31], hypoglycemic [32], anti-inflammatory [33], anti-bacterial [34], anti-ulcer [35], anti-arthritic [36], and immunomodulatory activity [26].

Alkaloids, tannins, polysaccharides, flavonoids, and phenolic compounds are among the biologically active compounds, each playing unique functional roles [34].

Researches have showed that the flavonoids and phenolic compounds found in caper can block the production of pro-inflammatory cytokines and chemokine, suggesting that caper has anti-inflammatory properties and might be used as a therapeutic approach for managing colitis [37,38]. Studies have showed that caper has immunomodulatory properties [34], which, besides its anti-oxidant and anti-inflammatory effects, could enhance its efficacy in treating colitis. In traditional medicine, caper's ethnopharmacological properties are effective in treating a range of ailments. Unfortunately, there is a lack of comprehensive research on the impact of the caper on treating UC (Zhu et al., 2021). In our recent study, we developed a hydro-alcoholic extract from the caper plant to investigate how it can protect against acetic acid-induced UC in rats. Our study looked at how the extract affected clinical symptoms, ulcer healing, and its anti-inflammatory and antioxidant properties.

## 2 Materials and methods

### 2.1 Plant material and extraction

The aerial parts of the caper were collected from the Kalat region in the Khorasan Razavi Province, Iran. Mrs. Sozani, a botanist from the School of Pharmacy at the Mashhad University of Medical Sciences in Mashhad, Iran, could identify the plant specimen. The specimen was deposited in the herbarium of the Ferdowsi University of Mashhad with the herbarium number 13063.

To prepare the caper aerial parts, they were air dried in a shaded area and subsequently ground into a fine powder. The extraction process was conducted using the maceration method. In summary, 100 g of the dried powdered aerial parts were soaked in a 70 % v/v hydro-ethanolic solution for 72 h with intermittent shaking. The resulting mixture was filtered using Whatman No. 1 filter paper. After centrifugation at 3000 rpm for 5 min, the supernatant was concentrated using a rotary evaporator at 37 °C. Subsequently, freeze-drying was performed to remove the ethanol and water content and obtain a fine powder. The extract yield was 18.9 % w/w of the dry powder, which was stored at −80 °C until further use.

By basing the dosages on previous research [[39], [40], [41]], two different concentrations of caper extract were used: 100 mg/kg and 300 mg/kg. To achieve these concentrations, 1 g or 3 g of caper powder was added to 4 mL of vehicle containing polysorbate 80 (Tween 80) and 2 mL of dimethyl sulfoxide (DMSO), respectively. The solutions were then diluted to a final volume of 100 mL. For administration to the rats, 2 ml of the prepared solution was administered orally through gastric gavage using a nasogastric tube.

### 2.2 Chemicals and reagents

Nitric Oxide (NO) assay and Lipid Peroxidation (MDA) assay kits were provided by Sigma-Aldrich Chemical Co. (St. Louis, MO, USA). IL-6, IL-10, TNF-α ELISA kits and Glutathione (GSH) assay kit, were purchased from Zellbio (Germany). All other materials were analytical and standard, including: 5,5′-dithiobis-(2-nitrobenzoic acid) (DTNB), trichloroacetic acid (TCA), Thiobarbituric acid (TBA), pyrogallol, malondialdehyde (MDA), ethanol 96 %, Dimethyl sulfoxide (DMSO), TRIS-EDTA, ammonium chloride, hydrochloric acid (HCl), acetic acid, sulfasalazine 100, Prednisolone, and 3-(4,5-Dimethylthiazol-2-yl) (MTT) purchased from Sigma-Aldrich Chemical Co. (St. Louis, MO, USA).

### 2.3 Animals and ethics statement

Sixty adult male rats, aged 20 weeks and weighing between 200 and 250 g, were obtained from the Laboratory Animal Unit of Mashhad University of Medical Sciences (MUMS) to conduct the study. The rats were housed in individual cages, maintaining standard conditions of temperature (22–25 °C) and a 12-h light/12-h dark cycle. They were given continuous, unrestricted access to food and water for the entire experimental period. The animal experimentation procedures followed the ethical guidelines approved by the Animal Ethics Committee of Mashhad University of Medical Sciences, Mashhad, Iran (IR.MUMS.SP.1396.144). Adequate veterinary supervision and surveillance were implemented throughout the study to ensure the well-being of the animals and compliance the NIH guide for the care and use of laboratory animals (NIH Publication No. 80–23; revised 1978).

### 2.4 Colitis induction

Prior to our study, an evaluation was conducted to determine the optimal concentration of acetic acid for inducing colitis. Ten rats were administered varying concentrations of acetic acid, namely 2 %, 3 %, 5 %, and 8 %. Subsequently, multiple parameters, including macroscopic evaluation of the colon, weight changes, fecal consistency, rectal hemorrhage, and survival, were assessed. Based on the observations made, it was determined that the 3 % concentration of acetic acid exhibited the highest efficacy among all tested concentrations.

Following an accommodation period, except for the healthy control group, the experimental procedure begun by administering 3 % acetic acid on day zero with a 2 mm in diameter catheter under mild anesthesia [ketamine hydrochloride (100 mg/kg), xylazine (10 mg/kg), and acepromazine (5 mg/kg) mixture]. A catheter was inserted 6 cm into the anus, and 2 ml of acetic acid was administered. To prevent intra-colonic solution leakage, the rats were held in the inverted trendelenburg position during rectal installation and for 2 min thereafter.

To ensure an empty colon, the rats underwent a 24-h fasting period prior to colitis induction.

### 2.5 Experimental design and treatment administration

The animals were randomly assigned to six groups (n = 10 each) as described below.

Healthy control: lacking colitis induction and treatment (non-colitic control group).
Control colitis: with induced colitis only received vehicle (polysurbat 80 and DMSO 1 %).
Sulfasalazine-treated colitis: colitic animals treated with sulfasalazine at a dose of 100 mg/kg;
Prednisolone-treated colitis: colitic animals treated with prednisolone at a dose of 50 mg/kg
caper-treated colitis groups: colitic animals treated with caper at doses of 100 mg/kg and 300 mg/kg.

The administration of treatments was conducted orally through a nasogastric tube for a duration of 8 days, starting 24 h after the induction of colitis.

On the 8th day of the experiment, the rats were euthanized, and a section of the colon was collected for both biochemical and gross examination. Specifically, the distal one-third of the colon, extending proximally up to 7 cm from the anal orifice, was taken for analysis. The chemical doses used here were per previous studies [[39], [40], [41]].

### 2.6 Outcome measures

We quantified daily clinical progress using the disease activity index [42]. Throughout the study period, we also recorded the survival rates.

The colonic damage was assessed using the colon macroscopic scoring system [43,44].

The colonic fragments were analyzed for oxidative stress markers such as malondialdehyde (MDA), myeloperoxidase (MPO) activity, and nitric oxide (NO) tissue levels, as well as antioxidant stress markers including superoxide dismutase (SOD), catalase (CAT), and glutathione (GSH) levels. The study aimed to investigate how changes in inflammatory cytokine levels in colon tissues affect the underlying mechanisms.

#### 2.6.1 Clinical symptoms assessment

Progression and intensity of colitis were assessed daily by quantifying the Disease Activity Index (DAI) as described by Cooper et al. [42] with minor adjustments. The DAI is a composite measure that considers changes in body weight, fecal consistency, and rectal bleeding (Table 1).

**Table 1:** Scoring of the disease activity index (DAI).

Score	Decrease in growth %	Stool Consistency*	Occult/gross rectal bleeding
0	0	Normal	Normal
1	1∼5	Normal	Normal
2	5∼10	Loose stools	Normal
3	10∼15	Loose stools	starting bleeding from anus
4	>15	Diarrhea	Gross bleeding

* Normal stools: stools that are normal and consist of well-formed pellets.

Loose stools: the stools are semi-formed, loose, and do not adhere to the anus.

Diarrhea: loose, watery stools that adhere to the rectum.

DAI score is determined by summing the scores for each parameter in the following manner: DAI = (weight loss score) + (stool consistency score) + (hematochezia score).

#### 2.6.2 Macroscopic assessment

The rats were humanely euthanized using ether eight days following the administration of acetic acid. The colons, spanning from the rectum to the ileocecal valve, were carefully excised, longitudinally cut, and gently rinsed with a 0.9 % saline solution to remove any fecal material.

To assess the extent of mucosal damage, the colon macroscopic scoring system developed by Wallace et al. [44] was employed. The scoring system is presented in Table 2. The Wallace score is calculated as the sum of the scores for each criterion (ulceration, adhesion, bowel wall thickening). A blinded observer conducted all damage scoring without knowledge of the therapy. The scoring criteria considered “inflammation” to be hyperemia and thickening of the intestinal wall.

**Table 2:** Criteria for scoring of gross morphologic damage.

Score	Gross morphology
0	No damage
1	Localized hyperemia, but no ulcers or erosions
2	Localized hyperemia and thickening of bowel wall. No ulcers
3	One ulcer without thickening of the bowel wall
4	Two or more sites of ulceration or inflammation
5	Two or more major sites of ulceration and inflammation or one site of ulceration/inflammation extending > l cm along the length of the colon.
6–10	If damage covered >2 cm along the length of the colon, the score was increased by 1 for each additional centimeter of involvement.

#### 2.6.3 Biochemical measurement of colonic inflammation

Pre-weighted colonic fragments were homogenized in ice-cold phosphate-buffered saline (PBS, pH 7.4) to make 10 % w/v homogenate. Homogenization in MDA detection was performed using KCl 1.15 %. Homogenate was centrifuged at 2500 RPM for 10 min at 4 °C. Biomarkers of oxidative stress and the amount of cytokines in the colonic samples were measured by separating the supernatants from the hemogenized samples and using commercially available kits or spectrophotometric assays to test them.

### 2.7 Oxidative stress assay

#### 2.7.1 Lipid peroxidation assay

The lipid peroxidation assay was performed following the method described by Ohkawa et al. [45]. In this assay, a 10 % (w/v) tissue homogenate was combined with sodium dodecyl sulfate, acetate buffer (pH 3.5), and an aqueous solution of thiobarbituric acid. The mixture was then heated at 95 °C for 60 min, resulting in the production of a red pigment. This pigment was extracted using a mixture of n-butanol and pyridine, and its concentration was determined by measuring the absorbance at 532 nm. To establish a standard reference, tetramethoxy-propane was used, and the level of lipid peroxide was quantified in terms of nanomoles of malondialdehyde.

#### 2.7.2 SOD activity assay

SOD activity was measured using a spectrophotometer based on the Madesh method [46]. In this method, superoxide is produced through the autoxidation of pyrogallol, and the reduction of the tetrazolium dye MTT to its formazan by superoxide is inhibited. The inhibition level is quantified by measuring formazan absorbance at 570 nm. The addition of dimethyl sulfoxide (DMSO) ends the reaction and assists in the dissolution of the resultant formazan. The resulting color is stable for an extended period.

Each unit of SOD activity, expressed as units per milligram of protein, represents the protein amount required to reduce MTT by up to 50 %.

#### 2.7.3 Total thiol levels assay

To determine the total thiol level, we employed the method described by Sedlack and Lindsay [47]. This method involves the reaction of thiol groups with Ellman's reagent, or DTNB, resulting in the formation of a yellowish complex.

In this method, 50 μL of tissue homogenate were combined with 1 mL of 0.3 M Tris-EDTA buffer (pH = 2.8) in a 10-ml test tube. Subsequently, 20 mL of 0.01 M DNTB and 3.16 mL of absolute methanol were added to the mixture. The test tube was capped, and the color development occurred for 15–20 min. Afterward, the mixture was centrifuged at 3000 g for 10 min at room temperature. The absorbance of the resulting supernatant was measured at 412 nm, and the absorbance of a DTNB blank was subtracted from it. A blank containing the sample without DTNB was also included in the analysis.

#### 2.7.4 Catalase enzyme activity assay

Catalase enzyme activity was measured based on its ability to decompose hydrogen peroxide (H2O2) by the Aebi method [48]. The decomposition of hydrogen peroxide could be investigated by decreasing the absorbance at 240 nm. For this purpose, 30 % hydrogen peroxide was used as a substrate, and 50 mM phosphate buffer with pH = 7 was used as a substrate substitute in the blank solution. The reaction was started by adding hydrogen peroxide, and the decrease in absorbance was checked using a spectrophotometer at a wavelength of 240 nm for 3 min. Each unit of catalase activity is defined as the number of micromoles of hydrogen peroxide consumed per milligram of protein.

#### 2.7.5 Nitric oxide metabolites (nitrite) assay

To quantify the production level of nitric oxide (nitrite) metabolites, the supernatant obtained from the separation process was used. For the quantification of nitrite, a colorimetric method provided by the kit manufacturer was employed, as per their instructions. Briefly, the standard solution was prepared according to the manufacturer's instructions and subsequently diluted. A volume of 50 μl of the test samples was added to each well of the microplate, followed by the addition of 50 μl of the sulfonamide derivative solution to each well. The samples were then incubated at room temperature for 10 min. Subsequently, 50 μl of the NED solution was added to each well, and the samples were incubated again at room temperature for another 10 min. The absorbance of the samples was read at a wavelength of 550 nm (ODs).

### 2.8 Inflammatory cytokines assay

The enzyme-linked immunosorbent assay (ELISA) method was used to assess the cytokine production in colonic segments. ELISA kits from the German company Zellbio were used to assess the levels of TNF-, IL-6, and IL-10. The results of the ELISA test were reported as picograms per milliliter (pg/ml), and the procedure was performed under the manufacturer's instructions.

### 2.9 Data analysis

The collected data was analyzed using GraphPad Prism® 8 software. Parametric data were expressed as the mean and standard deviation (SD), while non-parametric data were presented as the median and interquartile range (IQR). To compare the groups, parametric data were analyzed using one-way ANOVA with Tukey's multiple comparisons post-hoc test. Non-parametric data were analyzed using the Kruskal-Wallis test, followed by Dunn's test for multiple comparisons between groups. The level of statistical significance was set at p < 0.05, showing a significant difference between the groups being compared. In this study, only the most meaningful significant findings are depicted in the figures to ensure clarity and focus on the key outcomes.

## 3 Results

### 3.1 Impact of caper on clinical symptom and macroscopic assessment of acute acetic acid-induced colitis

**Fig. 1:**
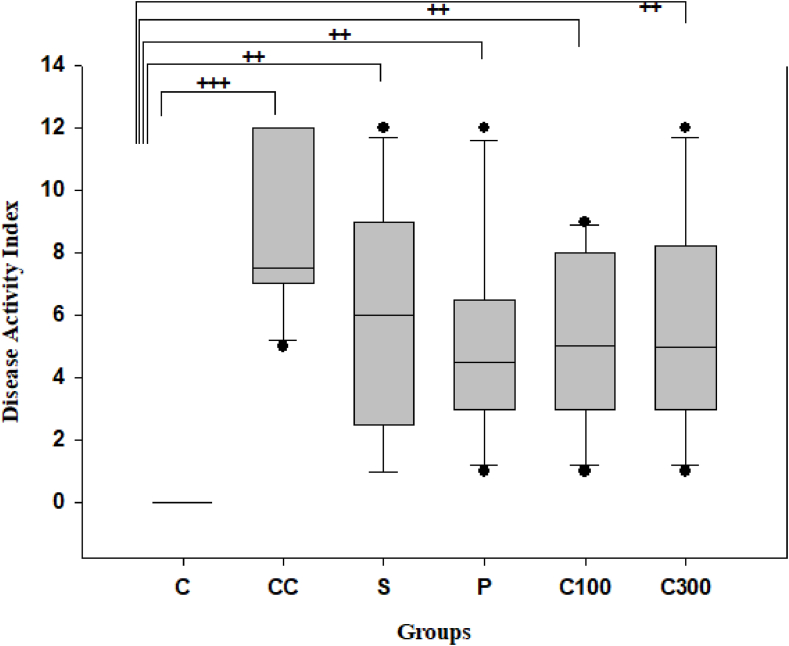
Disease activity index (DAI) in acetic acid-induced colitis model. **Legend:** Acetic Acid-induced growing disease activity index were assessed in C: control group, CC: control colitis, S: sulfasalazine -treated group, P: prednisolone--treated group, C100: 100 mg/kg of caper-treated group, C300: 300 mg/kg of caper-treated group; The statistical differences between groups were compared by Kruskal-Wallis Test and Dunn's Post Hoc Test. *p < 0.05, **p < 0.01, ***p < 0.001 vs. control colitis;+p < 0.05, ^++^p < 0.01, ^+++^p < 0.001 vs. control group.

The progression of disease in rats with acute colitis induced by acetic acid was evaluated using DAI and macroscopic disease severity score, compared to healthy control rats. There was no observable improvement in clinical symptoms of acute colitis in the colitic rats that received treatment after 8 days (p > 0.05) when compared to the control colitis group. Most of the rats exhibited a weight loss of approximately 4 % and also suffered from gastrointestinal issues like diarrhea and occasional rectal bleeding. In colitis rats, the DAI on day 8 resulted in a score of 12, whereas healthy control rats showed no symptoms and had a DAI score of approximately 0 (p < 0.001) (Fig. 1). The rats with colitis who received treatment showed clear clinical symptoms of acute colitis compared to the healthy control group (p < 0.01).

**Fig. 2:**
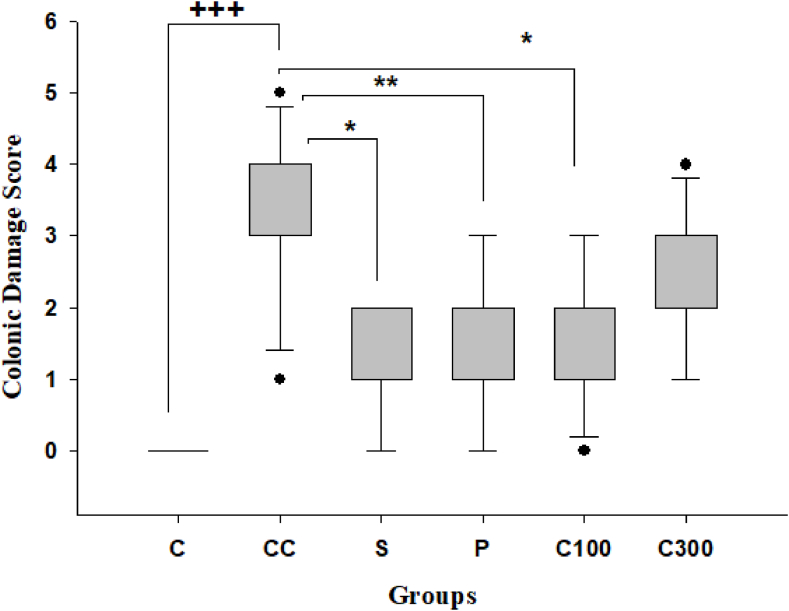
Colonic macroscopic damages in acetic acid -induced colitis model. **Legend:** Colon macroscopic damage score were assessed in C: control group, CC: control colitis, S: sulfasalazine -treated group, P: prednisolone--treated group, C100: 100 mg/kg of caper-treated group, C300: 300 mg/kg of caper-treated group; The statistical differences between groups were compared by Kruskal-Wallis Test and Dunn's Post Hoc Test. *p < 0.05, **p < 0.01, ***p < 0.001 vs. control colitis; + p < 0.05, ++p < 0.01, +++ p < 0.001 vs. control group.

The groups were compared, and it was found that there was a significant difference in the macroscopic colonic damage scores (ulceration, adhesion, bowel wall thickening) between the animals with colitis and the healthy control animals (p < 0.001) (Fig. 2). Compared to the control colitis group, both the sulfasalazine-treated and prednisolone-treated groups exhibited a substantial decrease in macroscopic colon damage (p < 0.05 and p < 0.01, respectively). Although the administration of 300 mg/kg of caper did not yield any notable effects, the group that received 100 mg/kg of caper showed a significant decrease in macroscopic colonic damage scores compared to the control colitis group (p < 0.01) (Fig. 2). Sulfasalazine, prednisolone, and different dosages of caper showed no notable variations.

**Fig. 3:**
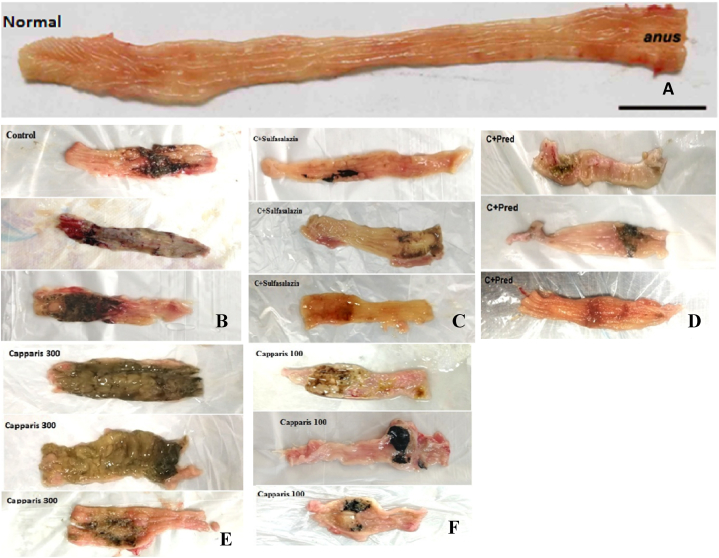
Macroscopic presentation of acetic acid-induced colitis in treatment groups; A) lacking colitis induction and treatment, they received normal saline) 2 ml, orally) which shows intact colon B) control colitis treated with vehicle (2 ml of polysurbat 80 and DMSO 1 %, orally) which shows the highest level of tissue injuries like edema, erythema, ulcer, necrosis and thickening of tissue C) colitic animals treated with sulfasalazine (100 mg/kg, orally) and D) colitic animals treated with prednisolone (50 mg/kg, orally) which both have shown significant improvement in ulcer healing and tissue damage; caper-treated colitis groups (E, F) with doses of 100 mg/kg showed significant improvement in ulcer healing and tissue damage (F).The extract did not exhibit the same level of effectiveness at a dose of 300 mg/kg (E).

At the end of the eighth day, the rats were sacrificed, and their colons were collected. Fig. 3 illustrates the overall appearance of the colonic luminal surface. The colon of the healthy control rats is unaffected and shows no evidence of tissue damage or inflammation (Fig. 3-A). Control colitis group exhibited the most severe tissue damage, characterized by swelling, redness, ulceration, tissue necrosis, and tissue thickening (Fig. 3-B). Our findings showed that colitis induced by acetic acid has resulted in severe damage. Treatment with both sulfasalazine (Fig. 3-C) and prednisolone (Fig. 3-D) effectively minimizes tissue damage and facilitates the healing of ulcers. Our research revealed that colitis induced by acetic acid led to significant tissue damage, which was not effectively alleviated by treatment with Capper 300 mg (Fig. 3-E).At a dosage of 100 mg/kg, the caper extract showed a notable enhancement in the healing of ulcers and reduction of tissue damage (Fig. 3-F).

### 3.2 Impact of caper on oxidative stress parameters in acute acetic acid-induced colitis

**Fig. 4:**
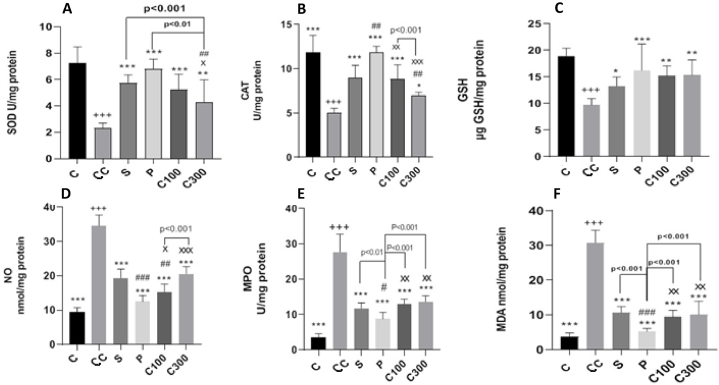
Effect of caper on enhancing antioxidant defenses in acetic acid-induced colitis in rats. **Legend:** Levels of the **(A)** Superoxide dismutase (SOD), **(B)** Catalase (CAT), **(C)** Total glutathione (GSH), **(D)** Nitric oxide (NO), **(E)** Myeloperoxidase (MPO) and **(F)** Malondialdehyde (MDA were measured in colonic tissues. Values were obtained from at least three independent experiments and are presented as the mean ± SD (n = 10/group). The statistical differences between groups were compared by one-way ANOVA with Tukey's multiple comparisons post-hoc test. *p < 0.05, **p < 0.01, ***p < 0.001 vs. control colitis;+p < 0.05, ^++^p < 0.01, ^+++^p < 0.001 vs. control group; #p < 0.05, ##p < 0.01, ###p < 0.001 vs. sulfasalazine-treated group; ^×^ p < 0.05, ^××^p < 0.01, ^×××^ p < 0.001 vs prednisolone-treated group. C: control group, CC: control colitis, S: sulfasalazine -treated group, P: prednisolone-treated group, C100: 100 mg/kg of caper-treated group, C300: 300 mg/kg of caper-treated group.

SOD activity in the control colitis group decreased significantly (p < 0.001) compared to the control group, while the treated colitic groups showed an increase in SOD activity compared to the control colitis (p < 0.001). The group treated with caper at a dosage of 300 mg/kg showed a significant decrease in SOD activity compared to both the group treated with sulfasalazine (p < 0.001) and the group treated with prednisolone (p < 0.01) (Fig. 4-A).

Although there was a notable decline in catalase (CAT) activity in the control colitis group compared to the control group (p < 0.001), there was a significant rise in CAT activity in the sulfasalazine-treated (p < 0.001), prednisolone-treated (p < 0.001), caper 100 mg/kg -treated (p < 0.001), and caper 300 mg/kg -treated (p < 0.05) groups compared to the control colitis group. Prednisolone treatment resulted in a significant increase in CAT compared to sulfasalazine (p < 0.01) and both doses of caper when comparing the treated groups. When comparing the groups treated with caper at doses of 100 mg/kg and 300 mg/kg, it was found that the dose of 100 mg/kg had significantly stronger effects (p < 0.001) (Fig. 4-B). The CAT levels in the caper-treated group at 300 mg/kg were significantly higher than those in the control colitis group. The effectiveness of this treatment was lower than sulfasalazine (p < 0.01), prednisolone (p < 0.001), and caper 100 mg/kg (p < 0.001).

Levels of glutathione (GSH) in the control colitis group were significantly reduced compared to the control group (p < 0.001). Although the GHS increase exhibited significant differences in the sulfasalazine (p < 0.05), prednisolone (p < 0.001), caper 100 mg/kg (p < 0.01), and caper 300 mg/kg (p < 0.001) treated groups when compared to the control colitis group, there were no other significant differences observed between all treated groups (Fig. 4-C).

The control colitis group exhibited a significant increase in nitric oxide (NO) levels compared to the control group, with a statistically significant difference (p < 0.001). Compared to the control colitis group, all treatment groups exhibited significantly lower levels of NO (p < 0.001). The prednisolone-treated group exhibited a significantly greater decrease in NO levels compared to the sulfasalazine-treated group (p < 0.001), as well as the groups treated with caper at 100 mg/kg (p < 0.05) and 300 mg/kg (p < 0.001) (Fig. 4-D). Using caper at a dosage of 100 mg/kg proved to be more effective in reducing NO levels compared to treatment with sulfasalazine (p < 0.01)The group that received a 100 mg/kg dosage of caper exhibited a notable reduction in NO levels compared to the group that received a 300 mg/kg dosage of caper (p < 0.001).

The control colitis group showed a notable rise in myeloperoxidase (MPO) levels compared to the control group, with a significant statistical difference (p < 0.001). The MPO levels were significantly reduced in all treatment groups compared to the control colitis group (p < 0.001). Prednisolone treatment resulted in a significantly greater reduction in MPO levels compared to sulfasalazine (p < 0.01) and both doses of caper (p < 0.01). Both doses of caper had a similar effect in reducing MPO levels, and this effect was comparable to the treatment with sulfasalazine (Fig. 4-E).

The control colitis group exhibited a significant increase in MDA levels compared to the control group, as shown in Fig. 4-F. However, there was a notable decrease in the MDA levels observed in all treatment groups (p < 0.001). The reduction in MDA levels was similar for both doses of caper, and this reduction was comparable to that achieved with sulfasalazine treatment. Using prednisolone for treatment resulted in a significantly higher reduction in MDA levels when compared to sulfasalazine (p < 0.001) and both doses of caper (p < 0.01).

### 3.3 Immunomodulatory impact of caper in acute acetic acid-induced colitis

**Fig. 5:**
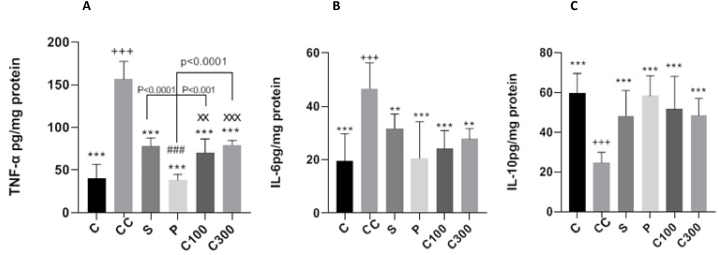
Effect of caper on colon cytokine production in acetic acid -induced colitis model. **Legend:** Protein levels of cytokines including (A) TNF-α, (B) IL-6 and (C) IL-10 levels in colonic homogenate were determined by ELISA in triplicate. Values are expressed as mean ± SD (n = 10/group). The statistical differences between groups were compared by one-way ANOVA with Tukey's multiple comparisons post-hoc test. *p < 0.05, **p < 0.01, ***p < 0.001 vs. control colitis;+p < 0.05, ++p < 0.01, +++p < 0.001 vs. control group; #p < 0.05, ##p < 0.01, ###p < 0.001 vs. sulfasalazine-treated group; ^×^ p < 0.05, ^××^p < 0.01, ^×××^ p < 0.001 vs prednisolone-treated group. C: control group, CC: control colitis, S: sulfasalazine -treated group, P: prednisolone-treated group, C100: 100 mg/kg of caper-treated group, C300: 300 mg/kg of caper-treated group.

The TNF-α levels were significantly increased in the control colitis when compared with the control group (p < 0.001). Compared to the control colitis group, all treatment groups exhibited a significant reduction in TNF-α levels (p < 0.001) (Fig. 5-A). Prednisolone treatment led to a significant decrease in TNF-α levels compared to sulfasalazine (p < 0.001), caper at a dose of 100 mg/kg (p < 0.01), and caper at a dose of 300 mg/kg (p < 0.001). The reduction in TNF-α levels was comparable between both doses of caper and the administration of sulfasalazine.

The control colitis group showed a significant rise in IL-6 levels compared to the control group (p < 0.001). While there were significant differences in IL-6 reduction between the sulfasalazine (p < 0.01), prednisolone (p < 0.001), 100 mg/kg caper (p < 0.001), and 300 mg/kg caper (p < 0.01) treated groups and the control colitis group, no other significant differences were observed among all treated groups (Fig. 5-B).

The levels of IL-10 showed a significant decrease in the control colitis group compared to the control group (p < 0.001). The levels of IL-10 showed significant differences in the sulfasalazine, prednisolone, 100 mg/kg caper, and 300 mg/kg caper treated groups when compared to the colitic control group (p < 0.001). There were no other significant differences observed among all the treated groups (Fig. 5-C).

## 4 Discussion

The present investigation shown that the hydro-alcoholic extract of caper (*Capparis spinosa* L.) had potential therapeutic benefits for ulcerative colitis (UC) in rats. This is achieved by modifying oxidative stress and inflammation through the enhancement of antioxidant enzyme activities and the reduction of inflammatory cytokines.

The study findings indicate that there were no significant differences in the clinical symptoms of colitis, such as weight loss, diarrhea, and rectal bleeding, among the various groups based on the Disease Activity Index (DAI) scores. Nevertheless, discrepancies in the Wallace score indicate noticeable variations in the extent of tissue damage in the colons of rats among the different groups. The difference in the DAI and Wallace score outcomes implies that although the treatments did not show visible effects on clinical symptoms, they might have influenced the structural integrity of the colon, as evidenced by the variations in the Wallace scores. These findings highlight the significance of assessing both clinical symptoms and structural changes in colitis models in order to fully evaluate the effects of treatment on disease progression. The variations in the Wallace score suggest that specific treatments may have had a greater impact on reducing colon tissue damage, emphasizing the need for additional studies on how treatments affect colon tissue damage to discover potential new treatment options for managing colitis.

By increasing antioxidant enzyme activities and reducing inflammatory cytokines, the hydro-alcoholic extract of the caper showed its ability to ease oxidative stress and inflammation. Further efforts should be made to isolate and identify the active compounds and constituents in caper for the treatment of UC, aiming to discover effective and innovative medications.

Our findings showed a decrease in anti-oxidant biomarker levels in rats with ulcerative colitis compared to healthy control rats, suggesting that UC has a detrimental impact on the body. Caper treatment, similar to standard treatments, effectively reduced oxidative stress by increasing the level of SOD, CAT, and GSH.

CAT is an enzyme that catalyzes the breakdown of hydrogen peroxide (H2O2) into oxygen and water. The level of CAT activity can show a cell's capacity to eliminate ROS and resistance to oxidative damage. The superoxide radical is dismutated (or partitioned) by SOD into either regular molecular oxygen (O2) or H2O2 (Hao & Liu, 2019). Glutathione Peroxidase (GSH-Px) is a key intracellular enzyme that converts H2O2 to water and lipid peroxides to their associated alcohols, primarily in the mitochondria but also in the cytosol. GSH-Px catalyzes the hydroperoxides by oxidating the reduced form of GSH into a disulfide form (Ighodaro & Akinloye, 2018; Marín-García, 2014). Studies have shown that a reduction in these antioxidant enzymes leads to oxidative stress, cell injury, and inflammation [49]. Consistent with previous research, our study discovered that the hydro-alcoholic extract of the caper effectively reduced oxidative stress by enhancing the activities of antioxidant enzymes (Tripathi et al., 2021b).

In our research, we observed increased levels of NO, MDA, and MPO after inducing UC. Using caper extracts, similar to conventional treatments, has been found to effectively decrease levels of oxidative stress. These findings suggest caper extract possesses both anti-inflammatory and antioxidant properties, which can play a role in preventing the advancement of diseases.

In animal models of IBD, increased production of ROS and reactive nitrogen species (RNS), such as superoxide, hydrogen peroxide, nitric oxide, hydroxyl radicals and peroxynitrite in the epithelium of colon is strongly linked with UC symptom severity [50]. It is worth noting that inflammation and oxidative stress can impact each other. Studies have shown that ROS triggers the activation of transcription factors like NF-κB, activator protein-1, and a protein kinase C family member, which worsen the inflammation [[50], [51], [52]].

NO, synthesized by NOS from l-arginine, regulates cellular processes like inflammation and oxidative stress by diffusing across cell membranes. High NO levels can cause nitrosative stress, tissue dysfunction, and vascular inflammation, while reacting with superoxide radicals to form peroxynitrite, further damaging cells and tissues [53,54]. Research shows that active ulcerative colitis typically results in an elevated production of NO, which decreases when treated with steroids [55,56].

MDA, a lipid peroxidation biomarker, is elevated in plasma levels in IBD patients, particularly Crohn's disease individuals, because of its role in cyclooxygenase from thromboxane synthesis [57,58]. Our findings showed a significant increase in MDA levels in rats with UC, but the administration of caper, similar to standard medication, effectively reduced MDA levels. This shows the success of caper in preventing lipid peroxidation and subsequent oxidative damage caused by the process.

MPO, an enzyme released by neutrophils, monocytes, and some macrophages, produces hypochlorous acid, a potent oxidant, causing oxidative stress, inflammation, and tissue damage [49,59]. The colonic mucosa of UC patients has shown higher levels of MPO, which the researcher believes might be a contributing factor to the disease's development (Masoodi et al., 2012). Our findings showed that the levels of MPO were significantly elevated in rats that had been induced with UC, and that caper, which is equivalent to commonly used treatment, was effective to lower MPO levels.

Furthermore, our findings revealed that caper-treated rats displayed significant decreases in TNF-α and IL-6 levels, while experiencing an increase in IL-10 levels, compared to a control group with colitis. This shows that caper has the potential to regulate inflammation in colitis.

IBD patients with elevated IL-6 levels produce acute-phase proteins like C-reactive protein, which correlates with poor clinical presentations and colon mucosal inflammation [[60], [61], [62], [63]]. TNF-α, as mentioned earlier exacerbates inflammation through different mechanisms including activation of NF-κB and upregulation of additional pro-inflammatory cytokines like IL-6 and IL-1β [64]. In our research, we observed a significant reduction in IL-6 and TNF-α - two important inflammatory cytokines - in the groups treated with caper, which was nearly comparable to the effects of conventional treatments (sulfasalazine and prednisolone).

Studies indicate a higher IBD risk in patients deficient in IL-10, an anti-inflammatory cytokine that suppresses the production of pro-inflammatory cytokines like TNF-α and IL-1 [65]. Additionally, the use of caper, sulfasalazine, and prednisolone in treatment led to elevated IL-10 levels and the stimulation of an immune-regulatory reaction. Overall, the study results show that caper has anti-inflammatory properties similar to the standard therapy for ulcerative colitis. This characteristic helps to reduce the inflammation that contributes to the progression of UC and induces a more tolerant immune response.

Our findings are consistent with earlier studies that have showed the efficacy of caper in managing inflammation, colitis, and stomach ulcers. They found caper could ease UC through the reduction of inflammatory cytokines and oxidative stress, which is in line with our findings as well.

In summary, the findings obtained in this study provide crucial evidence that the caper can provide innovative therapeutic approaches for treating ulcerative colitis. This is achieved by reducing inflammation and oxidative stress, which are fundamental elements of the pathogenesis of UC. However, our study faces a few limitations which can be considered for future studies. It failed to thoroughly examine the specific mechanisms responsible for its ability to treat ulcers and reduce inflammation, instead focusing on the overall impact without identifying any specific active compounds. Furthermore, the study did not examine the extract's long-term effects or safety profile, and the acetic acid-induced UC model may not accurately represent the pathology of human UC.

## 5 Conclusions

In conclusion, hydro-alcoholic caper extract showed a promising protective effect on treating acetic acid-induced UC in rat models via suppression of pro-inflammatory cytokine release and enhancing anti-oxidant activities. Our findings suggest that hydro-alcoholic caper extract can be a potential option for natural therapeutic products that can be used in the treatment of UC. However further studies are needed to better find out the mechanism and active products involved in its anti-ulcer and anti-inflammatory features.

## Ethics approval

This research was completed after receiving an ethical authorization code: IR.MUMS.SP.1396.144, from the Ethical Committee of Mashhad University of Medical Sciences.

Ethical approval for this study was obtained from the Mashhad University of Medical Sciences Ethics Committee.

## Funding

The Vice-Chancellor for Research of 10.13039/501100004748Mashhad University of Medical Sciences, Mashhad, Iran, supported this work financially.

## Data availability

The data sets used and/or analyzed during the current study are available from the corresponding author on request.

## CRediT authorship contribution statement

**Faezeh sadat hosseiniRavesh:** Writing – review & editing, Writing – original draft, Visualization, Project administration, Investigation, Formal analysis, Data curation. **AmirAli Moodi Ghalibaf:** Writing – review & editing, Writing – original draft, Visualization, Validation, Project administration, Formal analysis, Data curation. **Vahid Reza Askari:** Writing – review & editing, Writing – original draft, Validation, Investigation, Formal analysis, Data curation. **Farzad Fayedeh:** Writing – review & editing, Writing – original draft, Validation, Investigation, Formal analysis, Data curation. **Vafa Baradaran Rahimi:** Writing – review & editing, Writing – original draft, Validation, Formal analysis, Data curation. **Leila Etemad:** Writing – review & editing, Writing – original draft, Validation, Investigation, Formal analysis, Data curation. **Zhila Taherzadeh:** Writing – review & editing, Writing – original draft, Validation, Supervision, Resources, Methodology, Funding acquisition, Formal analysis, Conceptualization.

## Declaration of competing interest

The authors declare that they have no known competing financial interests or personal relationships that could have appeared to influence the work reported in this paper.
